# 

**Published:** 1823-09

**Authors:** 


### MONTHLY CATALOGUE OF MEDICAL BOOKS.

*** *It having been hinted to us that gentlemen, sending copies of their Works, prefer having their titles given at length, it is our intention, in future, to comply with this suggestion: but it is to be observed, that no books can be entered on this list except those sent to us for the purpose;—in the lists hitherto transmitted, we find that the names of works have frequently been given as published, which have not appeared for weeks, or even months after.*

Correspondence and Communications addressed to his Majesty's principal Secretary of State for the Home Department, concerning the Introduction of Tread-Mills into Prisons; with other Matters connected with the Subject of Prison-Discipline. By Sir JOHN COX HIPPISLEY, Bart. D.C.L. F.R. & A.S.; a Bencher of the Inner Temple.—Nicol, London, 1823.

The Utility and Importance of Fumigating Baths illustrated; or a Series of Facts and Remarks, showing the Origin, Progress, and final Establishment (by order of the French Government), of the Practice of Fumigations for the Cure of various Diseases of the Joints, Paralytic Affections, Gout, Rheumatism, Bilious and Nervous Disorders, all Complaints of long standing, and Diseases of the Skin. By JONATHAN GREEN, Member of the Royal College of Surgeons, London, and late Surgeon in his Majesty's Navy.—Burgess and Hill, London, 1823.

Lectures on the Operative Surgery of the Eye: being the Substance of that Part of the Author's Course of Lectures of the Principles and Practice of Surgery which relates to the Diseases of that Organ; published for the purpose of bringing the Management of these Complaints within the Principles which regulate the Practice of Surgery in general. By G. J. GUTHRIE, Deputy Inspector of Hospitals during the Peninsular War; Surgeon to the Royal Westminster Infirmary for Diseases of the Eye; Consulting Surgeon to the Western Dispensary for the Diseases of Women and Children, and Assistant Surgeon to the Westminster Hospital; Lecturer on Surgery.—Burgess and Hill, London.

Anatomical Diagrams of Abstruse Parts of the Human Body. No. I. By G. D. DERMOTT, M.R.C.S.—Burgess and Hill, London.

### NOTICES TO CORRESPONDENTS.

*In our Review of* Mr. AVERILL's *Work, we have inadvertently fallen into an error, which we hasten to correct. We have said that* Sir ASTLEY COOPER *was not the first who tied the carotid artery; and so far we were correct. Nevertheless, Mr. Averill's accuracy remains unimpeached; for Sir Astley undoubtedly first performed that operation* for the cure of aneurism, *as Mr. A. has stated.*

